# Silicon uptake by a pasture grass experiencing simulated grazing is greatest under elevated precipitation

**DOI:** 10.1186/s12898-018-0208-6

**Published:** 2018-12-04

**Authors:** James M. W. Ryalls, Ben D. Moore, Scott N. Johnson

**Affiliations:** 1 Hawkesbury Institute for the Environment, Western Sydney University, Richmond, NSW Australia; 2 Centre for Agri-Environmental Research, School of Agriculture, Policy and Development, University of Reading, Reading, UK

**Keywords:** Clipping, Drought, Grasses, Silicon, Simulated herbivory, Water stress

## Abstract

**Background:**

Grasses are hyper-accumulators of silicon (Si) and often up-regulate Si following herbivory. Positive correlations exist between Si and plant water content, yet the extent to which Si uptake responses can be mediated by changes in soil water availability has rarely been studied and never, to our knowledge, under field conditions. We used field-based rain-exclusion shelters to investigate how simulated grazing (shoot clipping) and altered rainfall patterns (drought and elevated precipitation, representing 50% and 150% of ambient precipitation levels, respectively) affected initial patterns of root- and shoot-Si uptake in a native Australian grass (*Microlaena stipoides*) in Si-supplemented and untreated soils.

**Results:**

Si supplementation increased soil water retention under ambient and elevated precipitation but not under drought, although this had little effect on Si uptake and growth (tiller numbers or root biomass) of *M. stipoides*. Changes in rainfall patterns and clipping had strong individual effects on plant growth and Si uptake and storage, whereby clipping increased Si uptake by *M. stipoides* under all rainfall treatments but to the greatest extent under elevated precipitation. Moreover, above-ground–below-ground Si distribution only changed following elevated precipitation by decreasing the ratio of root:shoot Si concentrations.

**Conclusions:**

Results highlight the importance of soil water availability for Si uptake and suggest a role for both active and passive Si transport mechanisms. Such manipulative field studies may provide a more realistic insight into how grasses initially respond to herbivory in terms of Si-based defence under different environmental conditions.

**Electronic supplementary material:**

The online version of this article (10.1186/s12898-018-0208-6) contains supplementary material, which is available to authorized users.

## Background

In defending themselves against herbivores, plants can tolerate (e.g. via compensatory regrowth) and/or resist (e.g. via production of toxic chemicals or defensive structures) herbivory. Investment in defence requires resources that could otherwise be invested in growth or reproduction, and are thus costly to the plant. In order to mitigate these costs, many plant defences are induced in response to damage instead of being constitutively expressed [1, 2]. Many species of grasses, for example, are frequently defoliated by grazing ungulates, and often tolerate herbivory by replacing lost biomass. In comparison to woody plants, grasses make limited use of secondary metabolites and structural components to resist herbivores, however [3]. Phytoliths (i.e. microscopic deposits of silica, SiO_2_), in particular, confer defence against herbivores through abrasion on their mouthparts and diminished nutrient acquisition via reduced palatability and digestibility of foliage [4]. While grasses generally show fewer defences based on secondary metabolites than other plant taxa, Si may prime production of chemical defences, especially those associated with the jasmonic acid pathway (e.g. [5]).

Many grasses are hyper-accumulators of silicon (Si), sometimes accumulating up to 10% of their dry mass, more than any other inorganic constituent [6]. Their capacity for Si accumulation varies among species, however, with some species taking up more Si from the soil than predicted from the rate of transpiration (these are termed “active accumulators”) and others the same or even less (“passive” and “rejective” accumulators, respectively) [7, 8]. Si, as an energetically cheaper resource for plant structural support, may trade-off with carbon (C)-based defences [9]. Thus, plants that have high Si uptake typically have lower levels of C, and only recently has this been demonstrated in the roots [10]. Indeed, most studies have considered Si accumulation in the shoots, with little experimental work on how biotic and abiotic factors affect Si uptake and accumulation in the roots. This may provide only a partial insight because the majority of Si is often stored in the roots [11] and if Si is retained in the roots this would likely affect the extent to which Si accumulates in the shoots.

While changes in the Si-accumulating responses of grasses have been linked to grazing [2, 12], and have been associated with an active Si transport mechanism, others have identified no (e.g. [13]) or species-specific (e.g. [14]) Si uptake responses to grazing. Emerging evidence suggests that Si uptake can vary with soil Si availability, root and shoot biomass, and transpiration rate [15, 16], yet there is particular controversy over the extent to which induction responses can be mediated by changes in soil water availability and transpiration rate [17]. Moreover, as highlighted by SE Hartley and JL DeGabriel [4], we lack field-based data about how grasses accumulate Si, especially under different environmental conditions.

Plants can only acquire Si via uptake of silicic acid (Si(OH)_4_) from soil water by the roots [18]. Thus, decreases in soil water availability might be predicted to impair Si uptake. Increases in extreme precipitation events, including drought and elevated precipitation predicted for the future [19, 20], have a number of impacts on plant growth, physiology, nutrition and allocation to defences [21–23], yet we know relatively little about how water stress affects Si uptake and deposition, especially in non-crop plants. Some studies have shown that Si levels in grasses decline under drought [13, 24], whereas others suggest that grasses can maintain uptake [25, 26].

Using a field-based rain-exclusion experimental facility, this study aimed to determine how the growth and chemical characteristics of weeping grass (*Microlaena stipoides* Labill.) respond to water stress (drought and elevated precipitation), simulated grazing (i.e. clipping) and the application of Si to the soil, with a particular focus on changes in silicification of above- and below-ground tissues. *Microlaena stipoides* is common within grazed native pastures in the high rainfall zone (> 550 mm average annual rainfall) of south-eastern Australia [27] and is highly valued for its fodder quality, year round growth and resistance to drought. It is adapted to a wide variety of soil types and has been shown to accumulate high levels of shoot Si [28], making it an excellent candidate for testing Si uptake responses to water availability and grazing. Based on the high Si-accumulating capacity of grasses, their tendency to up-regulate Si in response to herbivory [12] and the importance of water availability in determining Si accumulation [13], we hypothesised that simulated grazing would increase Si uptake in *M. stipoides* roots and shoots, which would be enhanced by increased levels of precipitation. Moreover, Si might substitute for C as a cheaper plant structural resource [9], so increases in Si uptake were expected to coincide with decreases in C concentrations.

## Methods

### Rain-exclusion shelters

Rain-exclusion shelters (249 cm × 188 cm) located at the Hawkesbury campus of Western Sydney University near Richmond, NSW (latitude − 33.609396, longitude 150.737800), as described by Johnson et al. [29], were used to exclude 100% of ambient rainfall from four mesocosms beneath each of 18 shelters. Mesocosm pots (41 cm × 41 cm × 31 cm) were arranged in a 2 × 2 formation and dug into the ground so that the rim of the pot was flush with the soil surface. Each of the 72 mesocosms was filled with the excavated soil (chemical composition defined in Additional file 1: Table S1; methods from GE Rayment and DJ Lyons [30]), which was air-dried and sieved to < 4 mm.

### Experimental procedure

Six *Microlaena stipoides* (var. Burra) seeds were sown and grown in 288 individual seed cells (38 × 57 mm) under field conditions. After 4 weeks (on 26 September 2016), four cells were transplanted into each mesocosm pot. Shelters were assigned at random to one of three rainfall treatments giving six shelters per rainfall regime. The ambient treatment was set at 65 mm per month, which was the average precipitation in Richmond between September and November over the previous 30 years (Bureau of Meteorology, Australia). The drought and elevated precipitation treatments comprised 50% and 150% of the ambient rainfall amounts, respectively. This translated to an application of 586 mL (ambient precipitation), 293 mL (drought) and 878 mL (elevated precipitation) of rainfall water collected at the site three times per week. Soil moisture measurements were taken weekly using a 12 cm Hydrosense II probe (Campbell Scientific, Queensland, Australia).

**Fig. 1:**
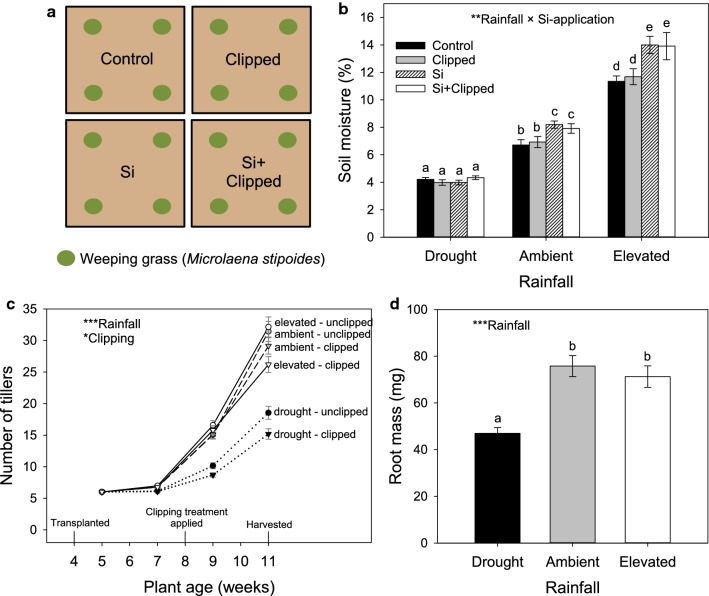
Schematic diagram of experimental plots beneath one rain-exclusion shelter (**a**). Significant treatment effects of rainfall, silicon application (Si) and simulated grazing (Clipping) on soil water content (**b**), the number of tillers (**c**) and root mass (**d**). Significance indicated by *(*P* < 0.05), **(*P* < 0.01), ***(*P* < 0.001). Values are means (± SE). Bars with the same letters were not significantly different (*P* < 0.05) according to Tukey's post hoc tests. Timeline of experimental events shown (**c**)

Each of two of the four mesocosms underneath each shelter were supplemented with Si by dissolving 146.5 mg of sodium metasilicate (Na_2_SiO_3_.9H_2_O) into the water applied under each watering regime. Four weeks after transplantation, grasses in two of the four mesocosms (one with Si applied and one without) were clipped, using hand shears, to leave approximately 40% of their aboveground biomass. The factorial treatment design under each shelter therefore comprised one mesocosm with Si applied alone (Si), one mesocosm with clipped grasses alone (Clipped), one mesocosm with both treatments applied (Si + Clipped), and one mesocosm with neither (Control; Fig. 1a). Plant heights and tiller numbers were recorded every 2 weeks throughout the experiment and whole plants were harvested 7 weeks after transplanting (when plants were 11 weeks old). Soil samples were taken from the centre of each mesocosm at a depth of approximately 5 cm and oven-dried. Roots were separated from shoots and frozen at − 20 °C, then oven-dried and weighed prior to chemical analysis.

### Chemical analyses

Dried root and shoot material and soil samples were ball-milled to a fine powder. Plant material from each mesocosm was pooled to provide enough dried material to conduct chemical analyses, giving one root and shoot sample per mesocosm (i.e. 72 roots and 72 shoots). Carbon (C) and nitrogen (N) concentrations were determined with a Carlo Erba CE1110 elemental analyser and total Si concentrations (% of dry soil mass) were determined with an X-ray fluorescence spectrometer (Epsilon-3x, PANalytical, EA Almelo, The Netherlands), as described in I Hiltpold, L Demarta, SN Johnson, BD Moore, SA Power and C Mitchell [31]. The “uptake efficiency” of Si (mg Si per mg root mass) was calculated by dividing the total content of Si in foliar tissue (i.e. % Si × shoot mass) by the total root mass.

### Statistical analyses

The R statistical interface v3.3.3 [32] was used to conduct statistical analyses. Impacts of simulated grazing, Si-application and rainfall on plant and soil chemistry (C, N and Si concentrations) were analysed using general linear models (based on pooled plant samples for each mesocosm). Plant growth characteristics (height until clipped, root mass and tiller numbers) and soil moisture were analysed with mixed models in the *nlme* statistical package [33], with mesocosm number and time (date) as random effects to account for repeated measures. The fixed terms for all models included clipping (clipped and unclipped), Si-application (Si + and Si −) and rainfall (ambient precipitation, drought and elevated precipitation). Two-way and three-way interactions between these terms were included in the model. Pairwise comparisons of means for treatment and interaction effects were made with Tukey’s post hoc tests utilising the *glht* function in the *multcomp* package [34]. Where necessary, dependent variables were transformed before analysis (Table 1). Correlations between Si and C concentrations were determined using the function *cor.test* in the package *stats*.

**Table 1:** Soil and plant responses to rainfall, Si-application and simulated grazing (clipping) treatments from linear/mixed models

Response variable	Fig.	Rainfall		Si-application		Clipping		Rainfall × Si–application		Rainfall × Clipping		Si–application × Clipping		Rainfall × Si–application × Clipping	
		*F*_2,60_	*P*	*F*_1,60_	*P*	*F*_1,60_	*P*	*F*_2,60_	*P*	*F*_2,60_		*F*_1,60_		*F*_2,60_	
Soil characteristics															
Soil moisture	1B	364.80	*<* *0.001*	22.47	*<* *0.001*	0.04	0.850	6.87	*0.002*	0.03	0.970	0.04	0.833	0.44	0.646
Soil Si^b^	2A	3.08	0.053	59.63	*<* *0.001*	1.41	0.240	1.96	0.150	0.41	0.667	0.02	0.903	0.04	0.959
Soil C	–	2.66	0.078	2.41	0.125	0.55	0.461	0.10	0.908	0.28	0.760	0.01	0.984	0.97	0.386
Soil N^a^	–	2.37	0.102	0.01	0.952	0.05	0.819	0.16	0.856	0.77	0.468	0.01	0.927	1.06	0.352
Plant growth															
Height^a^	–	5.89	*0.005*	4.29	*0.043*	0.96	0.332	0.34	0.715	0.16	0.852	1.26	0.266	0.05	0.956
Tiller number	1C	43.95	*<* *0.001*	1.27	0.265	5.57	*0.022*	2.15	0.125	0.46	0.632	0.01	0.953	0.09	0.913
Root mass^a^	1D	9.33	*<* *0.001*	2.99	0.089	0.64	0.426	0.78	0.465	0.32	0.726	0.44	0.512	0.21	0.814
Plant chemistry															
Root Si	2B	4.02	*0.023*	0.01	0.972	7.28	*0.009*	0.24	0.789	0.01	0.998	0.07	0.796	0.04	0.960
Shoot Si	2B	40.78	*<* *0.001*	0.88	0.353	42.04	*<* *0.001*	2.83	0.066	2.30	0.109	0.01	0.963	0.41	0.664
Root/shoot Si^b^	2B	18.75	*<* *0.001*	1.44	0.235	12.42	*<* *0.001*	1.85	0.166	0.02	0.979	0.01	0.992	0.02	0.984
Root C	2C	7.60	*<* *0.001*	0.13	0.720	5.86	*0.019*	0.34	0.714	0.32	0.725	0.01	0.953	0.25	0.780
Shoot C	2C	32.31	*<* *0.001*	0.03	0.862	52.14	*<* *0.001*	2.76	0.072	2.14	0.127	0.07	0.788	0.08	0.920
Root N	–	31.58	*<* *0.001*	0.01	0.934	3.77	0.057	0.08	0.923	1.29	0.282	0.37	0.545	0.35	0.706
Shoot N^c^	–	1.72	0.187	0.07	0.796	0.99	0.323	2.89	0.063	2.74	0.072	0.21	0.651	0.09	0.918

P-values highlighted in italic indicate significance (*P* < 0.05). Where appropriate, response variables were transformed (^a^log, ^b^square root, ^c^exp) before analysis. Height represents the height over time before the clipping treatment was applied

## Results

### Soil moisture and plant growth

Soil moisture was significantly affected by the interaction between rainfall and Si-application (Table 1), whereby soil moisture retention increased when Si was applied to the soil, but only under ambient and elevated precipitation conditions (Fig. 1b). Before the clipping treatment was applied, heights were significantly lower under drought compared with ambient and elevated precipitation and increased in Si-supplemented soils compared with untreated soils (Table 1). In general, plants under drought had significantly fewer tillers than those under ambient and elevated precipitation. Similarly, plants that were clipped had fewer tillers than those that were not clipped (Fig. 1c). Tiller numbers did not differ significantly between plants grown in Si-supplemented (13.18 ± 0.33) and untreated (12.62 ± 0.35) soil. Root mass decreased in plants subjected to drought, whereas root mass in plants under elevated precipitation did not vary significantly from those under ambient precipitation (Fig. 1d). Root mass was not significantly affected by clipping (clipped: 59.04 ± 2.50, unclipped: 70.18  ± 4.06) or Si-application (supplemented: 59.57 ± 2.97, untreated 64.65 ± 4.06). Full statistical results are shown in Table 1.

### Soil and plant chemistry

**Fig. 2:**
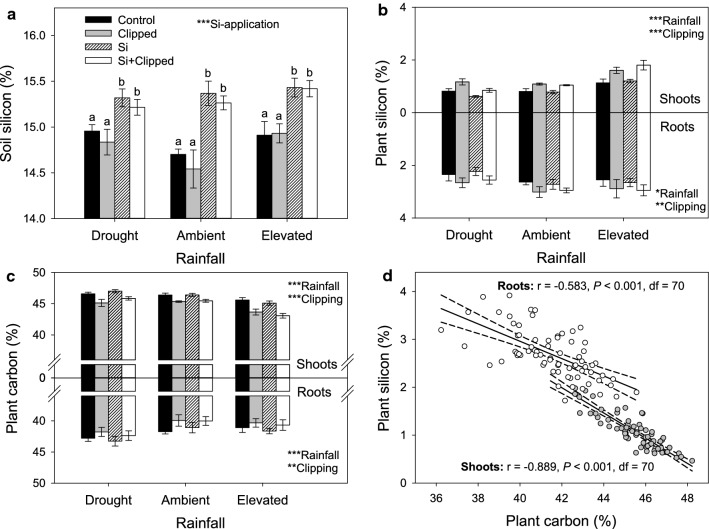
The effects of rainfall, silicon application (Si) and simulated grazing (Clipping) on soil silicon concentrations (**a**), *Microlaena stipoides* root and shoot silicon (**b**) and carbon (**c**) concentrations and negative correlations (± 95% CI) between concentrations of silicon and carbon (**d**). Significance indicated by *(*P* < 0.05), **(*P* < 0.01), ***(*P* < 0.001). Values of bars are means (± SE). Bars with the same letters were not significantly different (*P* < 0.05) according to Tukey's  post hoc tests

At the experiment’s conclusion, the Si concentration of Si-supplemented soil was significantly higher than soil that was not supplemented with Si (Fig. 2a), which likely reflected an increase in bioavailable silicon in the soil (c. 21 mg kg^−1^). The application of Si had no significant effect on the concentration of Si in the roots or shoots of *M. stipoides*, however. When plants were clipped, the concentrations of Si in the roots and shoots increased by 12 and 41%, respectively, compared with those that were not clipped (Fig. 2b). Grass roots and shoots tended to have higher concentrations of Si (Fig. 2b) and lower concentrations of C (Fig. 2c) under elevated precipitation compared with drought and ambient rainfall conditions, although these effects were less pronounced in the roots (Table 1). Root chemistry, in particular, responded more to drought than elevated precipitation, with Si and C concentrations decreasing and increasing, respectively, under drought compared with ambient rainfall. Concentrations of C and Si were negatively correlated (Fig. 2d). Moreover, Si concentrations in the roots relative to the shoots (i.e. the ratio of root to shoot Si) were significantly reduced in plants under elevated precipitation compared with those under ambient precipitation and drought. In other words, high water availability increased the uptake of Si in plant shoots relative to concentrations in their roots (Table 1). Rainfall had a significant effect on Si uptake efficiency (*F*_2,30_ = 39.83, *P* < 0.001), which increased by 84% under elevated precipitation (11.87 ± 0.75) and decreased by 40% under drought (3.89 ± 0.35) relative to ambient rainfall conditions (6.46 ± 0.76). In general, average soil moisture content was positively correlated with root (r = 0.25, *P* = 0.034, df = 70) and shoot Si concentrations (r = 0.61, *P* < 0.001, df = 70) and negatively correlated with root (r = − 0.26, *P* = 0.025, df = 70) and shoot C concentrations (r = 0.55, *P* < 0.001, df = 70). Root N concentrations increased and decreased under elevated precipitation (1.68 ± 0.03%) and drought (1.32 ± 0.03%), respectively, relative to ambient rainfall (1.53 ± 0.04%), whereas shoot N concentrations did not vary between treatments. Rainfall, Si-application and clipping had no significant effects on soil C or N concentrations (Table 1).

## Discussion

Only a handful of empirical studies [13, 35] have attempted to quantify the relative importance of biotic factors (e.g. grazing) and abiotic factors (e.g. water availability) on plant silicification, particularly in the field, as was recently highlighted by SE Hartley and JL DeGabriel [4]. The current study is one of the first experiments to manipulate precipitation regimes in relation to simulated grazing and Si uptake.

### Soil and plant responses to Si-application and water availability

The application of Si to the soil increased the retention of water in the soil, especially as rainfall increased, which was likely responsible for the increase in plant height in Si-supplemented plots observed before the clipping treatment was applied. The observed increase in soil water could be associated with either a physical effect of Si-application on the soil profile (via improved soil structure and water penetration; [36]) or an increase in plant water-use efficiency [37]. The application of Si in the form of Na_2_SiO_3_ could slightly increase soil concentrations of sodium (Na), though treatment concentrations were very low (1 mM) and field soil has the capacity to dissipate impacts. In any case, plants would most likely be unaffected as uptake of Na tends to decrease as plants take up Si [38].

Silicification of leaves has been shown to reduce water loss by as much as 30% in rice [39] and increase the biomass of drought-stressed wheat by 36% [40]. In this case, however, plant shoot and root Si concentrations did not respond to Si-application despite the increase in soil Si concentrations, suggesting that the uptake of plant-available H_4_SiO_4_ by *M. stipoides* may already be sufficient in the natural soil environment for optimal growth [41]. Under stressful conditions (e.g. grazing) or high water availability, however, the allocation of Si to structural defence or the passive uptake of Si, respectively, may increase, resulting in an increase in Si above the optimal level for plant growth [42].

Root and shoot Si concentrations and Si uptake efficiency were positively correlated with soil moisture, demonstrating the importance of plant water status in driving Si uptake and storage [13]. The accumulation of Si was associated with decreases in plant C concentrations, a trade-off response that has been identified in other grass species above- [43] and below-ground [10]. More specifically, however, the Si concentration of *M. stipoides* shoots relative to that of their roots was maintained under drought but was significantly higher under elevated precipitation, which was also evident in plants that had been clipped. The maintenance of shoot Si deposition under drought may be caused by or responsible for the drought tolerance that *M. stipoides* is generally known for [44], likely associated with osmotic adjustment and nutrient regulation [45]. Other grass species have been shown to maintain [25] or reduce foliar Si uptake under drought [24, 46]. Species-specific differences may be associated with changes in life-history strategies coupled with costs associated with Si accumulation [9]. Alternatively, the benefits of improved drought tolerance associated with Si accumulation in some species may outweigh the relative importance in others [47].

### The effects of simulated grazing on plant growth and chemistry

Silicification patterns in roots are understudied, despite evidence that Si concentrations can occur at highest levels in these tissues [11]. This study quantified the short-term changes in Si dynamics in both above-ground and below-ground tissues. In terms of plant growth, the results demonstrated that simulated grazing decreased the number of tillers but had no effect on below-ground biomass. Therefore, no compensatory growth in response to clipping occurred. Instead, *M. stipoides* responded to clipping by storing Si in its roots and shoots, indicative of an induced defensive response, which was consistent across all watering regimes. Kindomihou et al. [14] also identified a Si-uptake response to simulated grazing in three of five tropical fodder grass species, whereas KM Quigley and TM Anderson [13] identified no effects of simulated grazing on Si accumulation in two Serengeti grasses, suggesting that Si uptake responses are contingent upon plant identity and/or grazing intensity. Grasses that respond to wounding by increasing Si uptake likely involve active processes that are regulated at the gene level, as demonstrated by E McLarnon, S McQueen-Mason, I Lenk and SE Hartley [16]. The effects of simulated grazing on C concentrations showed clear opposite responses to Si concentrations, which were correlated in both roots and shoots and were consistent across all rainfall treatments. Si clearly helps protect grasses from wounding but this investment in structural defence may limit C sequestration.

Simulated herbivory may not mimic natural damage exactly but it enables researchers to control the type, timing and intensity of damage with fewer confounding effects [48], which is especially useful in field systems such as this. Plants subjected to simulated grazing combined with the application of phagostimulants or natural herbivory by grazing vertebrates increase plant defensive responses more than those that are subjected to simulated grazing alone [4]. Moreover, studies with repeated defoliation events (i.e. continuous wounding) have been shown to increase the Si content of grasses compared with single defoliation events, with Si concentrations increasing by up to 400% in response to leaf damage [2] and persisting for several months ([16] and references therein). *Microlaena stipoides* may therefore show an even greater defence response in the form of higher grazing-induced uptake of Si when subjected to herbivore attack. High levels of rainfall may further enhance Si uptake by increasing transpiration overall [13], with implications for reducing palatability and digestibility by herbivores. Drought, however, is likely to reduce this defensive response to promote resource-conservation (i.e. water and nutrient-uptake) over investment in plant structural defence [49]. Above-ground–below-ground Si distribution only changed under elevated precipitation, under which shoot Si concentrations increased relative to root concentrations. Si may therefore remain in the roots until plants have enough water to enable the transfer of Si from the roots to the shoots.

## Conclusions

This study provides clear evidence that water availability influences Si-based responses to damage in plants under field conditions. In particular, Si uptake responses to simulated grazing were enhanced when soil water availability was not limited, which are likely linked to a combination of active and passive uptake mechanisms. Field-based approaches such as this are fundamental to our understanding of how biotic and abiotic factors contribute to silicification in plants [4]. Studies that further incorporate changes in grazing intensity of above- and below-ground tissues would significantly advance our understanding of plant Si dynamics and its ecological importance.

## Additional file

**Additional file 1: Table S1.** Chemical composition of excavated soil (N = 12) used in the experimental study.

## Abbreviations

C: carbon
N: nitrogen
Si: silicon
SiO_2_: silica
