# Supplementary material for: Silicon uptake by a pasture grass experiencing simulated grazing is greatest under elevated precipitation
Source: BMC Ecol. 2018 Dec 4;18:53. doi: 10.1186/s12898-018-0208-6 (PMC6280423; doi:10.1186/s12898-018-0208-6)
Supplement: Supplementary file 1 — Additional file 1: Table S1. Chemical composition of excavated soil (N = 12) used in the experimental study. [file 12898_2018_208_MOESM1_ESM.docx]

**Additional file**

**Table S1.** Chemical composition of excavated soil (N = 12) used in experimental study. Analysis conducted on oven dried (40ºC) soil sieved < 2mm by Environmental Analysis Laboratory, Southern Cross University, Australia. Specific methods described in Rayment and Lyons (2011).

| **Method** | **Nutrient** | **Units** | **Mean ± SE** |
| --- | --- | --- | --- |
| KCL | Ammonium Nitrogen | mg/kg | 3.75 ± 0.33 |
|  | Nitrate Nitrogen | mg/kg | 42.42 ± 2.54 |
|  | Sulphur | mg/kg | 5.88 ± 0.18 |
| Colwell | Phosphorous | mg/kg | 39.00 ± 5.30 |
|  | Potassium | mg/kg | 290.92 ± 13.26 |
| LECO IR Analyser | Total carbon | % | 1.03 ± 0.02 |
| 1:5 Water | Conductivity | dS/m | 0.100 ± 0.005 |
|  | pH level | pH | 5.70 ± 0.05 |
| DTPA | Copper | mg/kg | 12.31 ± 0.41 |
|  | Iron | mg/kg | 41.59 ± 1.89 |
|  | Manganese | mg/kg | 71.19 ± 1.56 |
|  | Zinc | mg/kg | 2.15 ± 0.15 |
| Exc. | Aluminium | meq/100g | 0.16 ± 0.02 |
|  | Calcium | meq/100g | 2.25 ± 0.11 |
|  | Magnesium | meq/100g | 0.60 ± 0.04 |
|  | Potassium | meq/100g | 0.57 ± 0.03 |
|  | Sodium | meq/100g | 0.073 ± 0.003 |
| CaCl_2_ | Boron | mg/kg | 0.36 ± 0.01 |
| XRF | Silicon | % | 14.85 ± 0.06 |
| Gravel | - | - | 5% |
| Depth | - | - | 0-10 cm |

Rayment, G.E. & Lyons, D.J. (2011) *Soil Chemical Methods - Australasia*. CSIRO Publishing, Collingwood, VIC.
